# Reading numbers is hard, and the difficulty is a syntactic one: a descriptive analysis of number-reading patterns in readers with and without dysnumeria

**DOI:** 10.1186/s41235-025-00694-7

**Published:** 2025-12-09

**Authors:** Noa Handelsman, Dror Dotan

**Affiliations:** Mathematical Thinking Lab, School of Education and School of Neuroscience, Tel-Aviv University, 30 Haim Levanon St., Ramat Aviv, 6997801 Tel Aviv, Israel

**Keywords:** Number transcoding, Dysnumeria, Number syntax

## Abstract

Number transcoding, the ability to convert digits to words and vice versa, is a critical skill in mathematical literacy and in everyday life. While transcoding is known to be difficult for children, it is unclear whether it challenges adults too, and if so, what the source of that difficulty is. Here, we analyzed the number reading performance of 172 neurotypical adults. Their mean error rate was 6.5%, considerably higher than that typically observed in word-reading tasks, indicating that transcoding is a relatively challenging task even in adulthood. To investigate the cognitive origin of this difficulty, we examined the error types produced by these participants, as well as by a second group of 51 adults with a number-reading deficit (dysnumeria, mean error rate of 28.7%). In both groups, most errors reflected corruptions of the number’s syntactic structure. Moreover, among adults with dysnumeria, the most common subtype was syntactic dysnumeria. These findings indicate that, as in children, the primary challenge in adult number reading lies in the processing of the number’s syntactic structure.

**Supplementary Information:**

The online version contains supplementary material available at 10.1186/s41235-025-00694-7.

## Introduction

Mathematical literacy relies on multiple foundations, the central one of which is numbers. Within the realm of numbers, the decimal system is a major achievement that took centuries to develop and is not trivial to learn (Cheung & Ansari, 2021; Shalit & Dotan, 2024). This system can represent any number using a small lexicon—ten digits and several number words—combined according to a small set of syntactic rules. For example, one set of syntactic rules reflects the place-value principle: the same digit takes different values and morpho-syntactic verbal forms depending on its position (e.g., 2 in 23 vs. 42, corresponding to *twenty* vs. *two*). Another rule is that the digit 0 functions as a placeholder with a null value and no verbal manifestation, e.g., in 403. Mastery of the decimal system requires conceptual knowledge, namely knowing the digits, the corresponding words, and the syntactic rules; and cognitive proficiency in converting multi-digit numbers automatically and effortlessly across three representational formats, or ‘codes’: digits, words, and quantity (Dehaene, 1992; Dehaene & Cohen, 1995; Dehaene et al., 2003). Converting between these codes—especially between the symbolic ones (digits and words), a process known as transcoding—is critical in mathematics. Adults engage in transcoding regularly, not only in academic disciplines but also in everyday life—e.g., when reading phone numbers, writing checks, or interpreting salaries. In children, the ability to transcode digit strings to words (432 → four hundred thirty-two, when reading numbers) or vice versa (writing numbers to dictation) was identified as a key predictor of arithmetic skills (Banfi et al., 2022; Habermann et al., 2020).

### Is transcoding hard?

Many studies have examined transcoding in detail and underscored its importance (Barrouillet et al., 2004; Cipolotti & Butterworth, 1995; Cohen & Dehaene, 1991; Delazer & Bartha, 2001; Deloche & Seron, 1982; Granà et al., 2003; McCloskey, 1992; Noel & Seron, 1993). A fundamental finding in this body of research was that transcoding skills are not easily acquired. Children aged 6–7 make many errors in transcoding tasks (Power & Dal Martello, 1990, 1997), and this difficulty often persists even in the third and fourth grades (Moura et al., 2013; Shalit & Dotan, 2024). However, whether these challenges are fully overcome by adulthood remains an open question. Existing data suggest a developmental component, at least in part: performance varies widely among children, from 7 to 59% errors at the top and bottom deciles in third-graders’ number reading (Shalit & Dotan, 2024), likely reflecting differing stages of cognitive maturation. Yet it remains unclear how this ability evolves beyond childhood. In other academic domains, such as word reading, early struggles typically give way to high proficiency; e.g., Hebrew-speaking adults make only 1.7% reading errors, compared to 11.2% among second graders (Friedmann & Gvion, 2003; Spektor, 2012). Whether number transcoding shows a similar improvement is unknown. Do adults eventually master transcoding as they do word reading, or does it remain disproportionately difficult? The present study addresses this by examining number reading in adults.

Another approach to assessing the difficulty of transcoding is to study adults with poor transcoding skills. Such difficulty often reflects a deficit in transcoding mechanisms, termed Dysnumeria (Dotan & Friedmann, 2018). There is extensive neuropsychological literature on dysnumeria, both as a developmental syndrome and as an acquired disorder following brain damage (Benavides-Varela et al., 2016; Cappelletti et al., 2005; Cipolotti et al., 1994; Cipolotti & Butterworth, 1995; Cohen et al., 1994; Cohen & Dehaene, 1991, 2000; Delazer et al., 1999; Dotan & Friedmann, 2018, 2019; Friedmann et al., 2021; Lochy et al., 2004; McCloskey et al., 1986; Noel & Seron, 1993, 1995; Power & Dal Martello, 1997; Zamarian et al., 2006). Such neuropsychological single-case studies have proved indispensable for identifying subtypes of dysnumeria and clarifying processes in number reading and writing. However, their scope is inherently limited: they examine only a small number of individuals with selective deficits. They lack large-scale sampling of individuals with dysnumeria, and they rarely involve systematic comparisons of typically developing adults to groups of people with dysnumeria. The present study addresses these gaps by doing both.

### Why is number transcoding hard?

To identify the origins of difficulty in transcoding, researchers have used one of two complementary approaches. One treats transcoding as a challenge of acquiring knowledge; the other treats it as a challenge of developing cognitive proficiency in a complex cognitive operation with multiple sub-processes.

The first approach considers transcoding as a challenge of learning a certain body of knowledge: the digits, the number words, and the syntactic rules (Barrouillet et al., 2004; Power & Dal Martello, 1997; Shalit & Dotan, 2024). Almost invariably, the focus of these studies was children at the stage of transcoding knowledge acquisition (late kindergarten to third and fourth grades). These studies typically analyze children’s errors, each error type pointing to a specific gap in transcoding knowledge that was not yet acquired. For example, if a child has not yet learned which digit corresponds with the word ‘six’, or is not yet fluent in this digit-word mapping, digit-substitution errors are likely, e.g., writing forty-six as 45 in dictation (Moura et al., 2013). Similarly, insufficient knowledge of syntactic rules yields syntactic errors. For example, not knowing the rules that dictate how to convert digits according to their decimal positions can lead to reading 203 as ‘two thousand and three’ (Shalit & Dotan, 2024). Learning these syntactic rules and applying them fluently takes time: even five-year-olds show some syntactic knowledge of numbers (Barrouillet et al., 2010), but the implementation of this knowledge in transcoding is still not fluent even at the fourth grade (Dotan & Dehaene, 2016; Shalit & Dotan, 2024). The predominance of syntactic errors shows that syntax is the main obstacle for children during transcoding (Cheung & Ansari, 2021; Moura et al., 2013; Shalit & Dotan, 2024). However, it is unknown whether syntax remains the main challenge in transcoding for adults too.

The second approach, which better fits the present study, considers transcoding as a challenge of proficiency in a complex and multi-phased cognitive process with multiple sub-processes. Cognitive models of transcoding describe these sub-processes and the information flow between them (Campbell & Clark, 1992; Cohen, 2023; Cohen & Dehaene, 1991; Dotan & Friedmann, 2018; Efodi-Klerman & Dotan, 2024; McCloskey et al., 1986). These processes are separate from those for non-number words (for a review of the dissociations between number reading and word reading, see Dotan & Friedmann, 2019; and several studies show such dissociations also for writing, e.g., Cipolotti et al., 1994; Fischer-Baum et al., 2018; Granà et al., 2003; Noel & Seron, 1995; Zamarian et al., 2006). While full transcoding models’ details are beyond the scope of the present study, the important thing to acknowledge is that they classify transcoding sub-processes along three dimensions: modality, stage, and compositionality. Modality distinguishes between cognitive processes that handle digits from those that handle number words—a central distinction in the well-accepted Triple Code Model (Dehaene, 1992; Dehaene & Cohen, 1995; Dehaene et al., 2003). Stage distinguishes between input and output processes. Compositionality, which is the most relevant for the present study, distinguishes lexical and syntactic processes. Lexical processes handle the identities of individual digits and number words. Syntactic processes handle the relations among the lexical elements, such as identifying the length of the digit string, handling irregular verbal structures (e.g., teen numbers, not saying the 0), assigning a correct lexical class to each number word (ones, tens, teens) according to its decimal position, etc.

### The present study

The present study aims to bridge the above-mentioned gaps and to explore how difficult transcoding is for adults and what causes that difficulty. We focused on digits-to-words conversion, as when reading aloud a digit string (123 → one hundred twenty-three). This is one of the two basic transcoding skills; the other, words-to-digits conversion (as in number dictation), was not examined here (but in the Discussion, we briefly report a recent study from our lab that examined words-to-digit conversion in adults). We tested two groups: neurotypical adults with no learning disorders, and adults with dysnumeria. A third group of neurotypical university students is described in supplementary materials. Neurotypical readers showed a high error rate, indicating that transcoding is difficult for adults. With regards to the origin of the difficulty, across all three groups syntactic errors were the most common, pointing to number syntax as the main challenge.

## Method

### Participants

The participants were 223 Hebrew-speaking adults, and the experiment was conducted in Hebrew. Recruitment was carried out by several dozens of MA students at Tel Aviv University, over several years, using their personal connections and social networks. All participants gave informed consent to participate in the study; for participants younger than 18, their parents too. The study was approved by Tel Aviv University’s Ethics Committee. There were two groups of participants: neurotypical readers and participants with dysnumeria. In the supplementary material (http://osf.io/kzw52), we provide all responses and detailed error coding for every participant, and we report the number-reading patterns of another group of 36 university students. Their patterns were similar to those of the typical readers but with lower error rates.

#### Typical readers

This group included 175 participants (120 females) who declared to have no difficulties with numbers or mathematics. Ages ranged from 21 to 73, mean = 33;7 (33 years 7 months), SD = 12;5. Three participants with very high error rates (> 22.5% errors; more than 200% of the interquartile range above the 75th percentile) were considered as outliers and consequently excluded from some analyses, as described below. They were excluded from computing the dysnumeria threshold, which was based on the typical readers as a control group.

#### Participants with dysnumeria

This group included 51 participants (31 females) who declared to have difficulties with mathematics or numbers, and had many errors when reading numbers aloud, significantly more errors than the typical readers (ages 16–64, mean = 32;2, SD = 10;6). Participants were classified as having dysnumeria if they made 18 or more errors (15.3%). This threshold was set using Crawford and Howell’s (1998) t test with one-tailed *p* = .05, relative to the typical readers (see Section “Number reading is hard”). The 51 participants in the dysnumeria group were 48 participants recruited as explained above, and the three outliers from the typical-readers group (i.e., the three outliers were included in both groups). Individuals who reported difficulties in mathematics but did not meet the error threshold were not included in the study.

### Stimuli and procedure

Participants read aloud 120 numbers, shown as strings of 3–6 digits: 20, 38, 52, and 10 numbers with 3, 4, 5, and 6 digits, respectively. Of these, 56 numbers contained the digit 0, and 22 had 1 in the tens position, yielding in a teen word. No number contained the same digit twice. Numbers were presented in random order (same order for all participants), without a comma separator between the thousand and hundred digits. Stimuli were displayed either on a screen or as A4 print outs (Times New Roman 16, double spaced).

Participants were instructed to say each number as a structured multi-digit verbal number, as opposed to digit-by-digit naming (e.g., 123 as “one hundred twenty-three”). Their responses were written down by the experimenter in real time, audio-recorded, and re-transcribed later by another experimenter. Each participant was tested once, individually, in a quiet room, either in person or via an online video meeting. Participants were not allowed to employ strategies such as using their finger to segment the digit string; they were instructed not to touch or point at the numbers. There was no time limit, and participants were allowed to take breaks.

### Error coding

When a participant provided both a correct response and an incorrect response (in any order), the trial was coded as incorrect. When the participant made more than one response, errors were aggregated across all responses. For example, if 23 was read as “forty-three… no, sorry, twenty-five”, both digits were coded as incorrect.

Errors were classified into three main types: substitution errors, order errors, and syntactic errors. These error types indicate malfunctions in different number reading sub-processes (Blanken et al., 1997; Cappelletti et al., 2005; Cipolotti & Butterworth, 1995; Cohen & Dehaene, 1991, 2000; Dehaene & Cohen, 1991; Deloche et al., 1987, 1994; Dotan & Friedmann, 2018, 2019; Friedmann et al., 2010; McCloskey et al., 1986).

#### Substitution errors

This classification was used when a digit 2–9 was substituted with another digit 1–9, except when 1 was in the tens position (e.g., 794 → *seven hundred and fifty-four*), or when participants explicitly stated that they didn’t know a digit (23 → *twenty something*). Duplication errors—when a digit was substituted into another digit that appeared elsewhere in the number (e.g., 234 → *two hundred and thirty-two*)—were also classified as substitutions. By contrast, substitutions of 0 into a non-0 digit (102 → *one hundred and thirty-two*) or vice versa (123 → *one hundred and three*), and substitutions into teens (30 → *thirteen*), were classified as syntactic errors, because the digits 0 and 1 are considered to be syntactic markers (Cohen & Dehaene, 1991).

#### Order errors

These are errors in the relative order of the digits 1–9, e.g., reading 123 as 132 or 213. We used this coding whenever the mismatch in relative digit order could be detected, even if the response was partial (e.g., 123 → *one hundred and thirty-something*) or mixed substitution and order errors (123 → 135). If only the order of 0 versus other digits changed (e.g., 2,034 → 2,304), the error was not coded as an order error but as a syntactic error (see Section “Syntactic errors”).

#### Syntactic errors

Syntactic errors are violations of the number’s syntactic structure, specifically, any error that creates a discrepancy between the target and the response with respect to the *number word frame* (Cohen & Dehaene, 1991; Dotan & Friedmann, 2018), the sequence of number-word lexical classes (ones, tens, teens) and the word thousand. There are several types of syntactic errors:

*Class error*: producing the correct digit with an incorrect decimal class (ones, tens, teens, hundreds, etc.). They include *decimal shifts*—cases in which the participant said the digit as if it was in a different decimal position (e.g., 230 → *two thousand and thirty*); and *teen errors*—substituting a teen word with a non-teen word (13 → *thirty*) or vice versa (30 → *thirteen*). Decimal shifts were further subdivided into shifts in the first (leftmost) digit(s) of the number (2305 → 20,305, 23,005, or 235) and shifts in other positions (e.g., 2305 → 2035).

*Thousand error*: the word *thousand* was said incorrectly—omitted (e.g., 23,456 → *twenty-three*, *four hundred and fifty-six*), said in an incorrect position (234,500 → *two hundred thousand thirty-four and five hundred*), said more than once (e.g., 234,000 → *two hundred thousand and thirty-four thousand*), or distorted morphologically (in 4-digit numbers in spoken Hebrew, “thousand” is a morphological suffix rather than a separate word, but sometimes participants used the separate-word form. See supplementary material for details about the Hebrew number system).

*Zero substitution*: a non-0 digit was substituted for 0 (e.g., 123 → 103) or vice versa. These are syntactic errors and not substitutions, as they disrupt the number word frame (205 → 245 involves adding the tens class, a clear disruption of the verbal structure).

*Decomposition*: the number was decomposed into separate verbal numbers (e.g., 2345 → *twenty-three*, *forty-five*), sometimes due to producing the word “zero” (103 → *one hundred zero three*).

## Results

### Typical readers

#### Number reading is hard

**Fig. 1:**
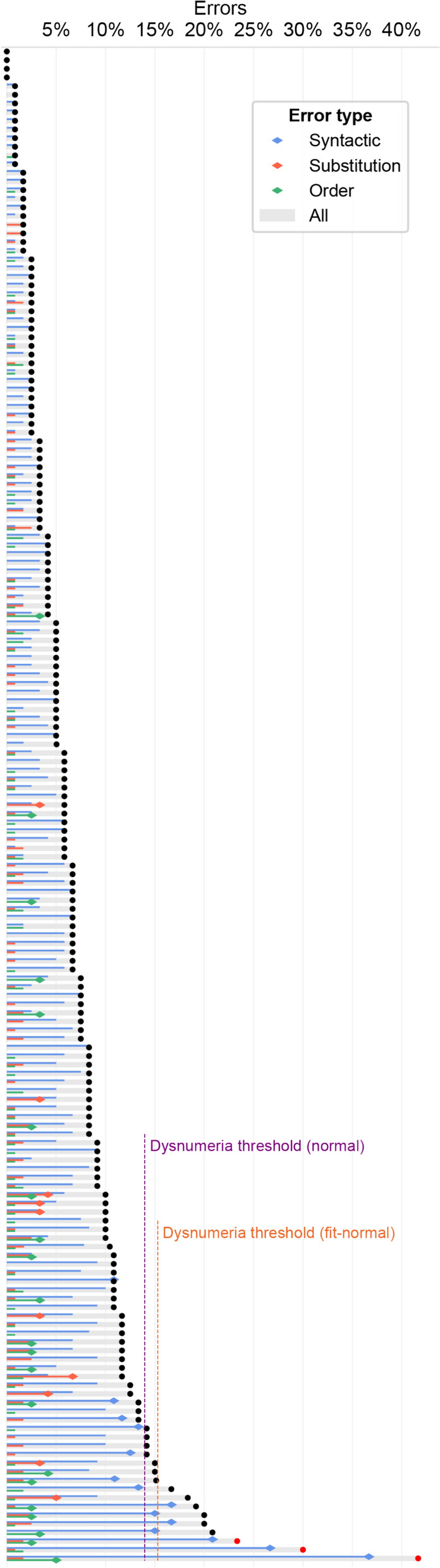
Number-reading errors among typical readers. Each participant’s overall error rate is marked by a grey bar with a black dot at its right end (red dot for the three outliers). Vertical dashed lines indicate dysnumeria thresholds: the purple threshold reflects Crawford and Howell’s (1998) one-tailed *p* ≤ .05 based on the group’s observed mean and standard deviation; the orange threshold is the same, but with corrected distribution parameters, (see text). Rates of specific error types are indicated by the internal colored bars; a dot at a bar’s right end denotes participants with significantly more errors than the group’s (Crawford and Howell’s one-tailed *p* ≤ .05, with corrected distribution parameters)

**Fig. 2:**
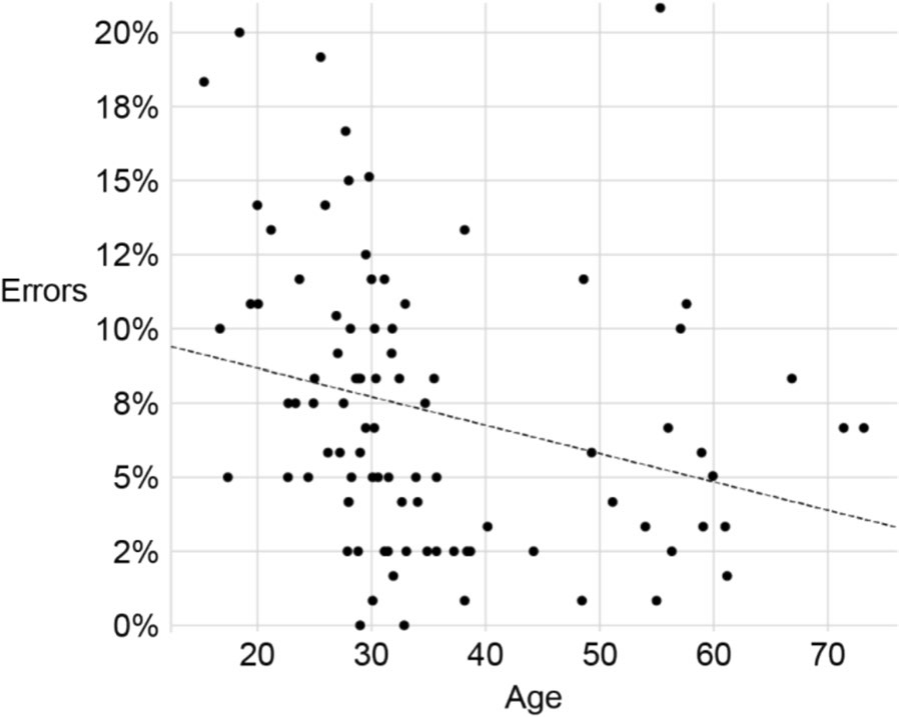
Error rates of the typical participants as a function of their age. The dashed line shows a linear fit, reflecting a small effect of age

The average error rate for the typical readers, excluding the 3 outliers, was 6.52% (SD = 4.48%, Fig. 1). Only four participants had no errors at all. The correlation between age and error rate was moderate but significant (excluding the three outliers, Spearman r = − .39, two-tailed *p* < .001, Fig. 2), such that older participants tended to make fewer errors.

These error rates reflect our coding scheme, which considered each erroneous response as an error even when accompanied by a correct response (e.g., immediate self-corrections). This approach is consistent with previous studies (Brock et al., 2017; Blanken et al., 1997; Dotan & Friedmann, 2018Gvion & Friedmann, 2016) and is well suited for detecting number-reading deficits, because both corrected and uncorrected errors indicate cognitive difficulty. When we considered only errors that were not self-corrected—a method less appropriate for diagnosing dysnumeria, but useful as a complementary index of performance level in real-word situations—the average error rate was 2.09% (SD = 2.17%).

#### What counts as dysnumeria?

**Fig. 3:**
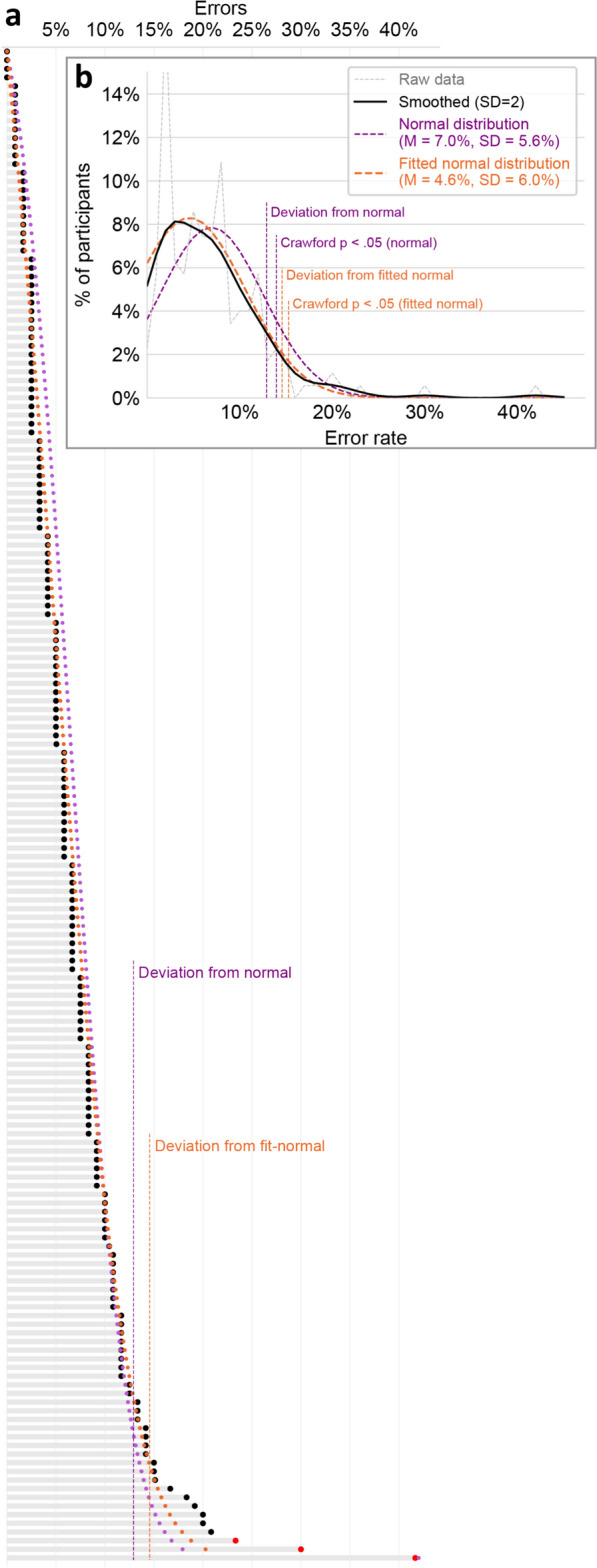
Typical readers’ error rates. **a** Per-participant overall error rates—same format as Fig. 1. Additionally, the purple and orange dots reflect the per-participant error rate predicted by a perfect normal distribution (same distributions as in panel **b**). The vertical dashed lines show the point at which the observed data deviates from these predicted values—i.e., participants with more errors than this deviation point consistently performed more poorly than a normal distribution’s prediction. **b** The distributions of errors. Thin grey line: histogram of the raw data. Black: same data, with Gaussian smoothing (σ = 2%). Purple: a normal distribution based on the observed mean and standard deviation. Orange: a normal distribution based on mean and standard deviation fitted to the data

We set the criterion for dysnumeria as having more errors than the threshold value that yields one-tailed *p* = .05 in Crawford and Howell’s (1998) t test compared to the typical readers (excluding outliers). According to the observed mean and standard deviation, this threshold is 17/120 errors (14.2%, dashed purple line in Fig. 1), and sixteen participants (9.1%, including the outliers) exceeded it. However, as shown in Fig. 3b, a normal distribution with these mean and standard deviation (purple curve) does not capture the data (black curve) very well. The reason for this deviance is that the observed data does not have a perfect bell-shaped normal distribution, but it is cropped at its left tail, because participants cannot make fewer than 0 errors. To accommodate this anomaly, we computed the mean (M) and standard deviation (SD) of a cropped normal distribution by fitting M and SD to the Gaussian-smoothed observed data (σ = 2%), with least squares as the fitting target function. The Fitted normal distribution (Fig. 3b, orange curve) seems to capture the observed data (black curve) quite remarkably. To look into this pattern more precisely, we computed each participant’s error rate as predicted by the fitted distribution. In this computation, we assumed that the per-participant error rates, transformed to percentiles, are equally spaced between 0 and 100% errors. The per-participant predicted error rates (orange dots in Fig. 3a) were virtually identical to the observed performance (black dots), except that the nine poorest-performing participants performed worse than the distribution’s prediction. In Fig. 3a, the dashed vertical line marks the point at which the observed error rate deviates from the one predicted by the distribution. This point of deviation from statistical normality could be used as another marker of dysnumeria, however, using it as a serious criterion for assessment would require, for the very least, a much larger sample, with more than nine participants to indicate the reliability of a statistical deviation.

We used a similar fitting procedure to compute the corrected normal distributions for each subtype of errors (Fig. S3). The results were essentially the same: for all error subtypes, the data were well modeled by a normal distribution cropped at 0%, and the fitted distribution captured the data better than the normal distribution based on the observed mean and standard deviation.

Given this, we argue that the better dysnumeria threshold is not the Crawford threshold computed from the raw mean and standard deviation, but a Crawford threshold computed from the fitted (orange) distribution. To compute this threshold, we corrected the *p* = .05 value: because 22.3% of the fitted distribution’s overall area is hidden below 0, a threshold of one-tailed *p* = .05 of the unhidden part of this distribution corresponds to *p* = .05 × (100% − 22.3%) = .039 of the whole normal distribution (including the hidden part). The threshold for this corrected one-tailed *p* is 15.3% (18/120 items), which is slightly more conservative than the standard threshold. Of the 175 participants who performed the experiment, all of whom declared to have no difficulties in number processing, nine participants (5.1%) exceeded the threshold and erred in 18 items or more, i.e., they might have dysnumeria. We highlight that this threshold of 15.3%, about 1 in 7 items, seems sufficiently high to entail a real-world difficulty, as expected for a number-reading disorder.

#### The main difficulty is processing the number’s syntax

**Fig. 4:**
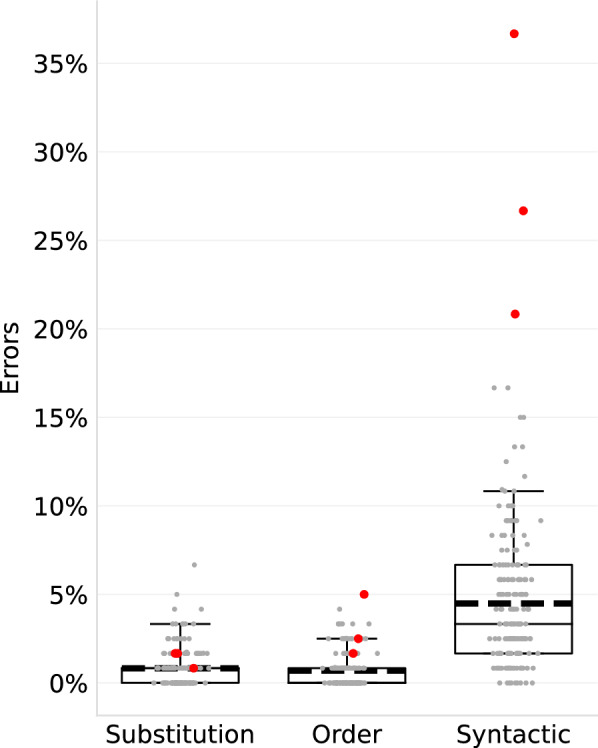
Distribution of error types in typical readers. The syntactic errors were the most frequent. The boxes show the 25th, 50th (median), and 75th percentiles; the whiskers show the 95th percentile; the thick dashed lines are the mean; and the dots show single participant data (red = the 3 outliers)

We classified the errors into syntactic errors, substitution errors, and order errors (Section “Error coding”). Most of the errors were syntactic (Fig. 4). There were more syntactic errors than substitution errors (paired t(171) = 13.87, two-tailed *p* < .001) and order errors (paired t(171) = 14.61 two-tailed *p* < 0.001). In line with findings from children (Moura et al., 2013; Shalit & Dotan, 2024; Steiner et al., 2021), for literate adults too, processing the syntactic structure of numbers poses a greater challenge than processing the identity of each digit or number word and their order. Within the syntactic errors, the most common subtype was first-digit decimal shifts (2.5% of all items), followed by errors involving ‘thousand’ (1.2%), and then by decimal shifts in non-initial digits (0.7%). Our data do not indicate the locus of these syntactic errors. They may originate from a difficulty at the visual input level—e.g., in detecting the number length or in parsing the digit string to triplets (Dotan & Friedmann, 2018), at the verbal production level—in creating the number’s verbal syntactic structure (number word frame, Dehaene & Cohen, 1991; Dotan & Friedmann, 2018; McCloskey et al., 1986), or in a core syntactic representation at an intermediate level (Dotan & Brutmann, 2022; Dotan et al., 2021a, 2021b; Friedmann et al., 2021; Handelsman & Dotan, 2023; McCloskey et al., 1986).

#### Triplet-based processing of the number

We examined the positions within each number where errors occurred. Syntactic errors often reflect a difficulty at the whole number level, but substitution and order errors can be analyzed per position.

**Fig. 5:**
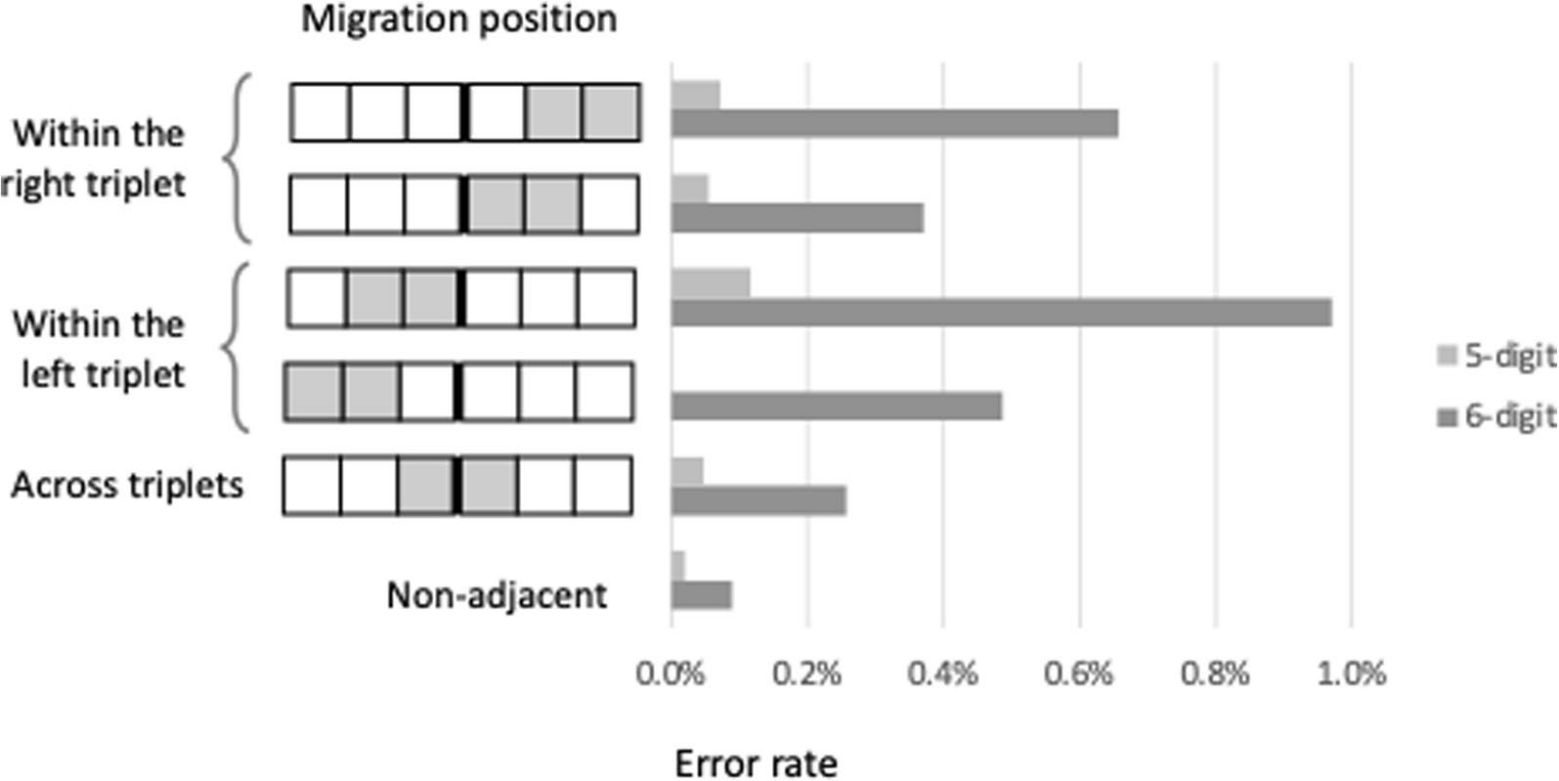
Percentage of order errors in 5-digit and 6-digit numbers in each position. There were significantly more migrations within triplets than across triplets

*Order errors*. We focused on digit migrations in 5- and 6- digit numbers, which are long enough to reveal possible division of the digits into triplets. Digit migrations to an adjacent location occurred in 0.2% of the 5- and 6-digit numbers, more frequently than non-adjacent migrations (0.04%, χ^2^(2) = 137, *p* < .001), so we focused on these exclusively. This adjacency effect has been observed in individuals with digit-order dysnumeria (Friedmann et al., 2010); here it characterizes typical reading too. Unsurprisingly, migrations were more frequent in 6-digit numbers (0.55%) than in 5-digit numbers (0.08%, χ^2^(2) = 147, *p* < .001), probably reflecting a two effects: first, a length effect (longer numbers are harder); second, the 6-digit numbers did not include 0, making adjacent migrations more probable (transpositions with 0 were coded as decimal errors, Section “Syntactic errors”). Migrations were also more frequent within a triplet than across triplets, even when we controlled for distance by considering only the adjacent migrations (Fig. 5; χ^2^(2) = 8.48, *p* < .001). This pattern suggests sensitivity to the triplet-based structure, in accordance with previous studies showing that both the visual (Dotan, 2022) and the verbal (Dotan & Brutmann, 2022) processing of numbers are triplet-based. The triplet-based structure may be a part of a multi-level hierarchical representation (McCloskey et al., 1986).

**Fig. 6:**
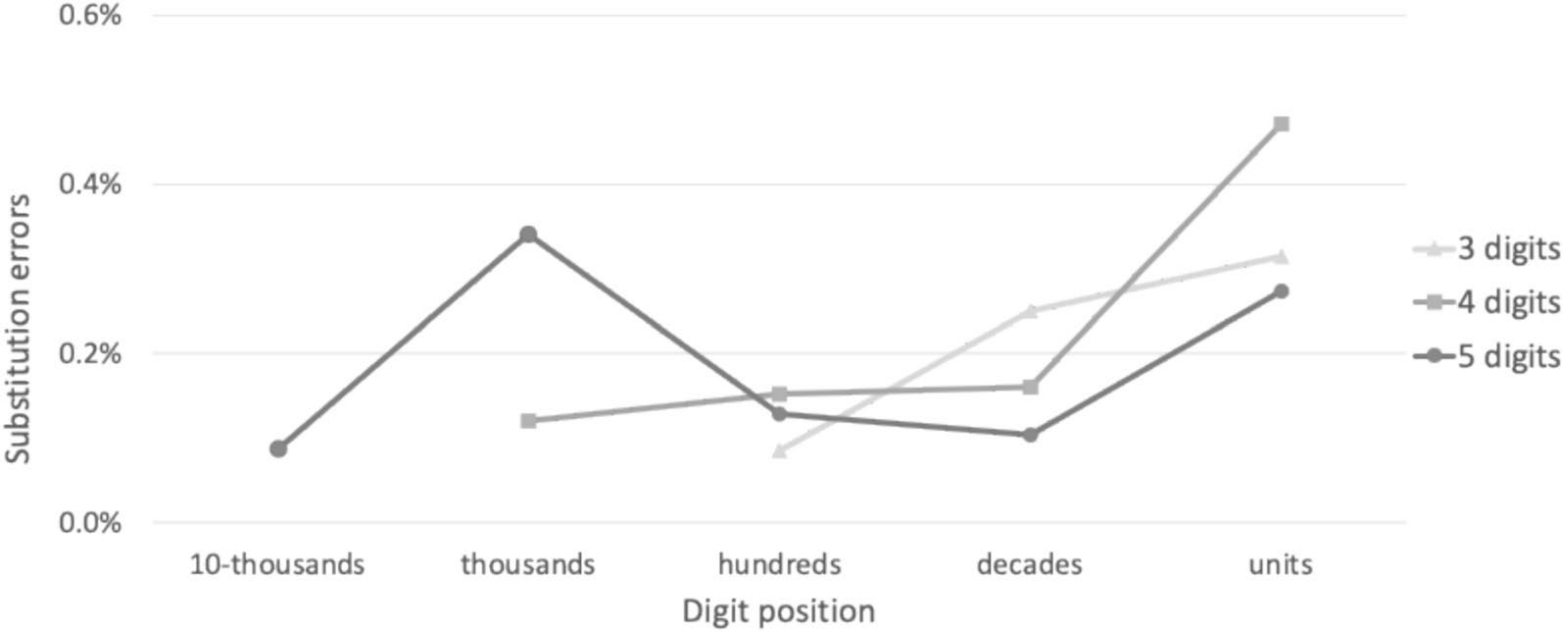
Substitution errors in each position. Error rates were counted out of the non-0 target digits in each position. Generally, the error rates increased for digits farther to the right end of the number. In 5-digit numbers, the error rate decreased from the thousands digit to the hundred digit, suggesting a chunk (triplet)-based processing, with the fewest errors in the beginning of a chunk and more errors thereafter

*Substitution errors*. In 3- and 4-digit numbers, the substitution error rate increased monotonically toward the right end of the number (Fig. 6). This pattern is in line with the idea that when reading numbers, the visual analyzer scans digits sequentially from left to right, so rightmost digits may get less resources (Dotan et al., 2021a, 2021b; Friedmann et al., 2010). In 5-digit numbers, the error rate decreased significantly between the thousand and hundred positions (1-tailed *t*(174) = 2.85, *p* = .004), again suggesting that long numbers (> 4 digits) are processed in a triplet-based manner. The beginning of a new triplet could improve performance for various reasons; for example, it may reset some resources, such as working memory and attention, and reallocate them to the new triplet. Future studies can examine precisely which mechanisms are involved in this process.

### Readers with dysnumeria

**Fig. 7:**
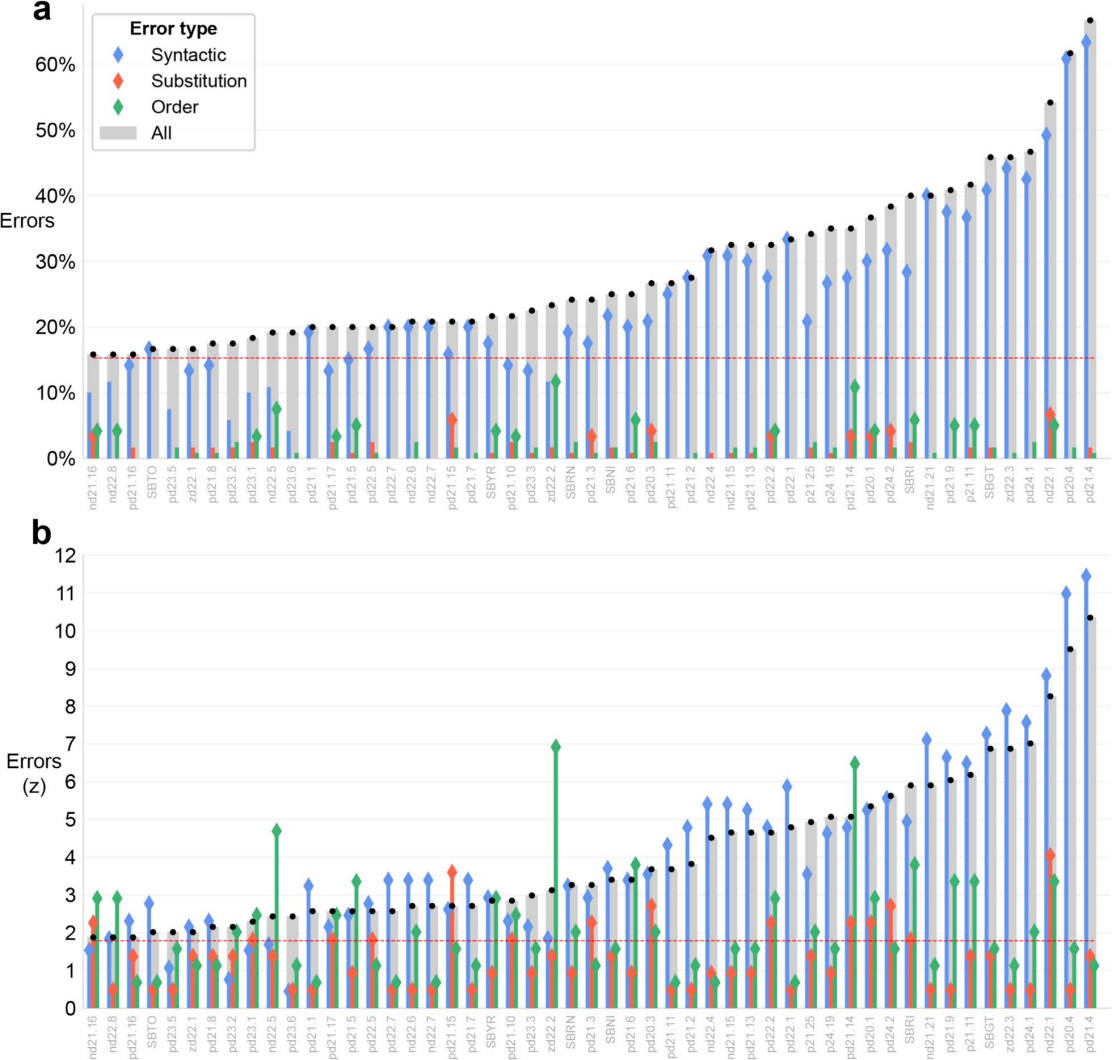
Error rates for participants with dysnumeria: **a** as percentages; **b** z-scored, computed separately for each error type, according to the typical readers’ mean and standard deviation. Each participant’s overall error rate is marked by a grey bar. Colored internal bars indicate rates of specific error types; a dot at the top of the bar denotes participants with many errors of that type (Crawford and Howell’s (1998) one-tailed *p* ≤ .05), indicating a type-specific dysnumeria. The dashed red line is the threshold for dysnumeria (18/120 errors). All thresholds and statistical comparisons are according to the corrected normal distribution (Section “What counts as dysnumeria?”)

The average error rate for the participants with dysnumeria was 28.7% (SD = 12.0%, Fig. 7a). As in the typical group, syntactic errors prevailed: all participants had more syntactic errors than substitution or order errors. Thus, for participants with dysnumeria too, syntactic structure posits the main difficulty when reading numbers. However, when we compared each error type to the typical readers (by z-scoring each error type separately) a few participants showed their most severe deficit was not syntactic but other (Fig. 7b).

**Fig. 8:**
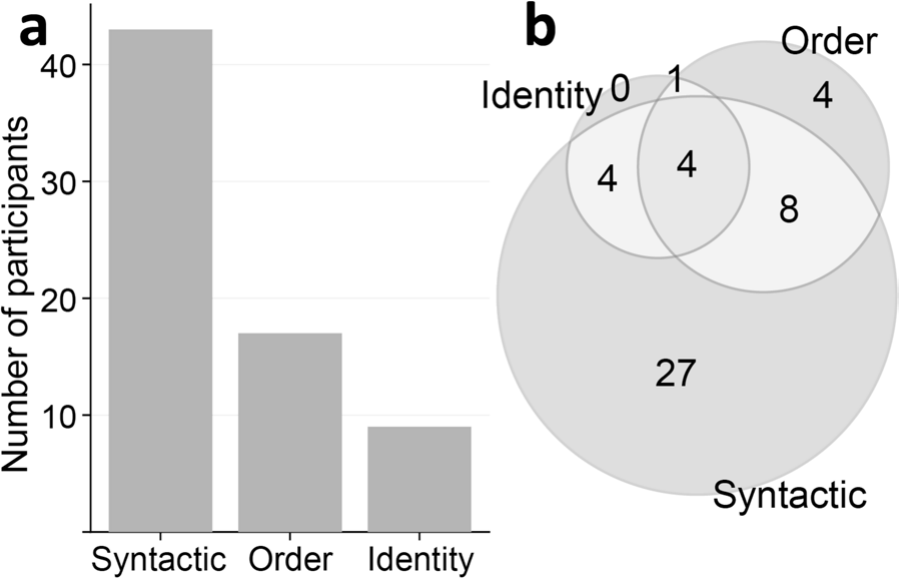
The number of participants having **a** each dysnumeria subtype and **b** each combination of subtypes. Overall, 17/51 participants showed multiple types of dysnumeria, and 3 participants did not show any type-specific dysnumeria

To obtain a clearer aggregate picture of participants’ specific difficulties, we classified each participant as having syntactic dysnumeria, digit-order dysnumeria, or digit/word-identity dysnumeria (several participants had multiple types). Thresholds for each type of dysnumeria were set using errors of that type, relative to typical readers, with Crawford and Howell’s (1998) t test with one-tailed *p* = .05, on the corrected normal distribution (see Section “What counts as dysnumeria?”). This resulted in a threshold of 15 syntactic errors (11.9%) for syntactic dysnumeria, 4 substitution errors (3.2%) for identity dysnumeria, and 4 order errors (3.0%) for digit-order dysnumeria. These three dysnumeria subtypes can be further subtyped (Dotan & Friedmann, 2018); however, diagnosing more specific subtypes requires additional number-processing tasks, which was not in the scope of the present study. Participants showed different dysnumeria profiles (some had multiple deficits), with syntactic dysnumeria being the most prevalent (Fig. 8).

## Discussion

### Transcoding is hard

The first goal of this study was to examine whether transcoding is difficult for adults. It is: our neurotypical adults showed a mean error rate of 6.5%, and 25% of them made more than 10% errors. Compared to word reading (mean error rate 1.7%, Friedmann & Gvion, 2003), another task that is considered basic and simple for educated adults, number reading is harder. Thus, our findings reveal that transcoding is either more cognitively challenging or less trained.

Unsurprisingly, the adults were better in transcoding than children in the third and fourth grades, whose average error rates on slightly easier numbers were 25.3% and 12%, respectively (Shalit & Dotan, 2024). Data from fifth graders, however, show performance on par with adults (Dotan et al., 2025). This suggests that the developmental trajectory ends around the fifth grade, and that at least some aspects of children’s difficulty are transient and do not persist into adulthood.

We examined number reading in a relatively large group of neurotypical adults, recruited from various sources to increase heterogeneity. We did not systematically test differences in number-reading skills across population subgroups, but our findings suggest they exist: typical readers averaged 6.5% errors, whereas the students’ group (see supplementary material) averaged 4.2%. This difference presumably reflects differences in education, socioeconomic status, personal skills, or other factors.

Our findings show that adults read numbers better than children and that number reading is harder than word reading, but the precise error rates depend on at least two methodological factors. First, the error coding method: we counted a response as incorrect even when participants self-corrected. This is a good method when assessing cognitive proficiency or dysnumeria, because self-corrected errors still indicate difficulty and can help reveal its source; it is also common in assessment of word-reading (Friedmann & Gvion, 2003). Second, the presentation format: our stimuli were numbers without comma separators at triplet boundaries (e.g., 12,345 and not 12,345), a method that increases the load on the visual analyzer of digit strings, and can consequently raise error rate (Dotan & Friedmann, 2018). This format improves sensitivity for detecting dysnumeria, but it may be overly pessimistic as an index of absolute performance in real-life situations, where numbers usually include commas.

The goal of this study was to obtain and characterize adult number-reading patterns in general, so we analyzed performance at the group level, and each participant was tested once. Future studies can investigate within-participant consistency and whether performance improves with practice. A recent study from our lab, which used a number dictation task, observed such a practice effect for individuals with dysnumeria (Cohen, 2023). Another open question concerns the potential effects of time pressure and the role of various strategies used by the participants.

### Syntax is the main origin of the difficulty

The main source of difficulty in number reading was processing numbers syntax. Two findings indicate this: first, syntactic errors were more frequent than non-syntactic errors in both typical and readers with dysnumeria. Second, among participants with dysnumeria, most (84%) had syntactic dysnumeria, i.e., a deficit in processing the number’s syntactic structure, whereas less than half had a non-syntactic dysnumeria.

These findings are in line with studies of transcoding in children. It takes children a long time to master syntactic skills such as understanding the role of different decimal positions (Cheung & Ansari, 2021) or handling irregular syntactic structures (Shalit & Dotan, 2024). This syntactic difficulty is in line with evidence that syntactic deficits are common also in language (Kesselman et al., 2018), and with the idea that syntactic processing is a complex, possibly human-specific, cognitive ability (Dehaene et al., 2015, 2022; Hauser et al., 2002).

Our analysis allowed for a rough classification of the dysnumeria cases into syntactic, order, and identity deficits, but we did not identify the specific locus of deficit for each participant. Our data cannot inform whether syntactic errors originated in the visual input stage, the core syntactic representation, or the verbal production stage, and it can certainly not inform which specific sub-processes within each of these stages are the origins of difficulty. Such a precise assessment requires deeper experimentation with additional tasks and finer grained analyses. For example, to determine whether syntactic errors originate at the visual input level, one would need to show poor performance on tasks that rely on visual input, such as same/different decision between pairs of numbers, and preferably, contrast this with good performance on tasks that do not require visual input (e.g., number repetition). To further show more specific deficits, one would need to use tasks tapping specific sub-processes. For example, to show that the visual-input deficit is at the process that detects the number length, the aforementioned same/different decision task must be used with appropriate stimuli: we must show high error rates in pairs of numbers that differ in length but not in digit identity or order (e.g., 6696–66,966), and better performance in pairs that do not rely on encoding length (e.g., same/different for 6696 vs. 6646). A detailed description of dysnumeria assessment tasks and protocols exceeds our focus here and will be described elsewhere.

Given the centrality of the syntactic challenge, it is informative to examine number transcoding and dysnumeria across languages, since verbal number systems and syntactic rules differ widely. In some languages, ones and tens are inverted: 54,321 would be said as ‘four-and-fifty thousand, three hundred one-and-twenty’. Such languages may require specific syntactic operations to handle inversion, and the prevalence and subtypes of dysnumeria may change accordingly. Indeed, symbolic numbers tasks show more errors in languages with decade-unit inversion (Clayton et al., 2020; Contreras-Saavedra et al., 2020; Ganayim et al., 2021), and the distribution of error types differs (Blanken et al., 1997; Proios et al., 2002). Some languages also mix base 10 and base 20; in French, 1–60 are base 10 and 61–99 are base 20, so 75 is literally ‘sixty and fifteen’. This irregularity is demanding and may affect typical performance as well as the prevalence and subtypes of dysnumeria (Pourquié & Nespoulous, 2018).

### Reading versus writing

The present study examined digits-to-words transcoding. A recent publication from our lab reported data of 79 neurotypical adults who performed the opposite task—words-to-digits transcoding (writing numbers to dictation; Efodi-Klerman & Dotan, 2024). Their results were similar to those obtained here: they made 4.6% errors on average (SD = 3.6%), showing that number dictation too is a challenging task. The somewhat better performance in dictation compared to reading could reflect inter-sample differences (see supplementary materials regarding inter-sample differences in the number reading task), or a genuine task difference. For example, dictation may pose a smaller cognitive load, and may afford different strategies compared to number reading—e.g., more opportunities for self-corrections because writing is typically slower than speaking.

As in our data, most number-dictation errors were syntactic: 61% of the number-dictation errors in Efodi-Klerman and Dotan, compared to 69% of the number-reading errors in our neurotypical sample. However, substitution errors were much more common in dictation (40% of the errors; absolute rate 1.8% of items) than in reading (12%; 0.8%). This likely reflects the heavier short-term memory load in dictation, which increases substitution errors.

### The definition and assessment of dysnumeria

The definition of dysnumeria makes it a unique a learning disorder, distinct from dyscalculia. Dyscalculia is an umbrella term that can refer to deficits in several numerical domains, including estimation, counting, transcoding, calculation, and arithmetic facts retrieval (Deloche et al., 1994; Gross-Tsur et al., 1996; Delazer et al., 2003; Proios et al., 2021; World Health Organization, 2019; American Psychiatric Association, 2013). Dysnumeria, on the other hand, is defined a specific disorder that disrupts number transcoding. The two disorders not only differ in definition, they also dissociate empirically: neuropsychological single-case studies show dissociations between dysnumeria and deficits in calculation or in the approximate number system. Moreover, dissociations exist among dysnumeria subtypes (Cohen & Dehaene, 1991; Dehaene & Cohen, 1991; Dotan & Friedmann, 2018; Dotan et al., 2014; Efodi-Klerman & Dotan, 2024; Handelsman & Dotan, 2023). These dissociations show the heterogeneity of learning disorders across domains within transcoding itself. Still, while considerable evidence indicates there exists a separation between dysnumeria and dyscalculia as well as between dysnumeria subtypes, separation does not necessarily entail independence. An interesting question for future studies is whether different mathematical cognitive disorders, including subtypes of dyscalculia and dysnumeria, tend to co-occur, and if so—why.

Given this heterogeneity of math-related disorders (also see Bartelet et al., 2014; Henik et al., 2015), assessment tools should include diagnostic tasks that detect specific disorders. Specifically with regards to dysnumeria, tools should assess both transcoding directions, i.e., number reading and writing, because these two tasks rely on separate cognitive processes that can be selectively impaired (Cipolotti et al., 1994; Cohen, 2023; Lochy et al., 2004). Several assessment tools have been developed to evaluate transcoding skills (Delazer et al., 2003; Karagiannakis & Noël, 2020; Semenza et al., 2014). To the best of our knowledge, the most comprehensive of these is the MAYIM battery developed in our lab (Dotan & Friedmann, 2014; Dotan et al., 2022).

We did not emphasize dysnumeria prevalence in this study because the distribution of individual error rates showed no clear bimodal separation between participants with and without dysnumeria. In the absence of such a natural division, as in many other learning disorders, we used a conventional statistical cutoff (α = .05), with a correction to accommodate the distribution’s shape. This approach often yields a circular outcome, with prevalence clustering around 5%. For these reasons, the key empirical finding is not the observed prevalence (which was indeed around 5%) but the dysnumeria threshold in our sample: 15.3% errors (one error in every 7 items). This is a high rate by any reasonable standard and clearly reflects a real-world difficulty, as should be the case when defining dysnumeria as a meaningful, academically relevant learning disorder.

## Conclusion

Although transcoding is a fundamental math skill and may seem trivial, it is surprisingly difficult. We showed that adults make many number-reading errors, mainly due to difficulty in processing the syntax of numbers. Given similar difficulties found in children (Cheung & Ansari, 2021; Shalit & Dotan, 2024), it may be a good idea to teach number reading explicitly in schools—something that, in many countries, is not done.

We also found that the prevalence of number reading dysnumeria is higher than expected under normal distribution, and that nearly all cases involve a syntactic deficit, either as a single deficit or accompanied by additional deficits in number reading. Basic assessment of dysnumeria is straightforward and can be done with a brief task like the one used here. We hope that drawing attention to this disorder, and to the ease of detecting it, will increase the way it is handled in educational systems.

## Supplementary Information

Additional file1 (DOCX 180 KB)

## Data Availability

The datasets generated and/or analysed during the current study are available in the Center of Open Science repository, http://osf.io/kzw52.
