# Supplementary material for: Reading numbers is hard, and the difficulty is a syntactic one: a descriptive analysis of number-reading patterns in readers with and without dysnumeria
Source: Cogn Res Princ Implic. 2025 Dec 9;10:84. doi: 10.1186/s41235-025-00694-7 (PMC12690019; doi:10.1186/s41235-025-00694-7)
Supplement: Supplementary file 1 — Additional file1 (DOCX 180 KB) [file 41235_2025_694_MOESM1_ESM.docx]

Supplementary material for

The syntactic origins of number-reading difficulties: a descriptive analysis of readers with and without dysnumeria

# The verbal number system in Hebrew

The syntax of Hebrew numbers is identical to English for numbers up to 100. Similar to English, there are number words for ones, teens, and tens (Table S1). The tens and teens words are derived from the ones words, in most cases with simple morphological modification (a minor phonological change and adding a suffix - /esre/ for teens, /im/ for tens). For example, 3 is /shalosh/, 30 is /shloshim/, and 13 is /shlosh-esre/.

Numbers larger than 100 are similar to English but with a few differences:

1. **Hundreds words.** Unlike English, in which the hundreds are said as a ones word + the decimal word “hundred”, in spoken Hebrew the hundreds are single words – a stem that reflects the digit, and a “hundreds” suffix (/-meot/). For example, the word for 3 is /shalosh/, and the word for 300 is /shloshmeot/. The English structure of “hundreds” with a 2-digit antecedent (“twenty-five hundred”) is ungrammatical in Hebrew.
2. **Thousands.** The structure of 5- and 6-digit numbers is similar to English – a 2- or 3-digit number followed by the word “thousand” and another 1- to 3-digit number. The only difference is that the Hebrew word for “thousand” (/elef/) is phonologically quite different from “thousands” (/alafim/). In contrast, 4-digit numbers have a completely different syntactic structure, which is similar to the structure of hundreds words: “thousand” is not a separate word but a morphological affix, and for some numbers the stem of the word is slightly different phonologically from the corresponding ones word; for example, 3 is /shalosh/ and 3,000 is /shloshtalafim/.
3. **200 and 2,000.** The standard Hebrew form for a hundreds word is /X-meot/, and for a thousand word (in a 4-digit number) it is /X-talafim/. However, for 200 and 2,000 Hebrew does not use the standard suffixes but a single-word irregular form – 200 is /mataim/ and 2,000 is /alpaim/.
4. **10,000** (did not appear in any stimulus). As explained above, Hebrew has a different syntactic structure for 4-digit numbers and for 5- and 6-digit numbers. The numbers between 10,000-10,999 are irregular because they do not use the 5-digit structure but the 4-digit structure – i.e., a morphological conjunction of the word “ten” with the “thousand” suffix. Thus, 10 is /eser/ and the 10,000 is /aseretalafim/.

The ones and teens words are inflected either to masculine or to feminine. When saying the number of objects, the number should agree with the object’s masculinity or femininity (which is often arbitrary). For arbitrary numbers, which do not count objects (such as in our experiment), Hebrew speakers usually use the masculine form for “one” (/exad/) and the feminine form for other numbers.

| **Table S1.** The phonological forms of Hebrew number words (feminine). | | | | | |
| --- | --- | --- | --- | --- | --- |
|  | **Ones** | **Teens** | **Tens** | **Hundreds** | **Thousands** |
| 1 | axat | axatesre | eser | mea | elef |
| 2 | ʃtayim | ʃtemesre | esrim | mataim | alpaim |
| 3 | ʃaloʃ | ʃloʃesre | ʃloʃim | ʃloʃmeot | ʃloʃtalafim |
| 4 | arba | arbaesre | arbaim | arbameot | arbatalafim |
| 5 | xameʃ | xameʃesre | xamiʃim | xameʃmeot | xameʃtalafim |
| 6 | ʃeʃ | ʃeʃesre | ʃiʃim | ʃeʃmeot | ʃestalafim |
| 7 | ʃeva | ʃvaesre | ʃivim | ʃvameot | ʃvatalafim |
| 8 | ʃmone | ʃmonaesre | ʃmonim | ʃmonameot | ʃmonatalafim |
| 9 | teʃa | tʃaesre | tiʃim | tʃameot | tʃatalafim |

#

# List of stimuli

| **Table S2.** The list of numbers the participants read. | | | | | | |
| --- | --- | --- | --- | --- | --- | --- |
| Block 1 | | |  | Block 2 | | |
| 825 | 3,916 | 25,816 |  | 36,749 | 70,246 | 7,418 |
| 2,730 | 50,682 | 4,259 |  | 97,415 | 518 | 207 |
| 589 | 379 | 75,826 |  | 65,324 | 9,053 | 9,314 |
| 43,806 | 70,425 | 90,236 |  | 708 | 89,456 | 615 |
| 916 | 6,498 | 5,034 |  | 537 | 53,097 | 63,918 |
| 803 | 35,097 | 9,408 |  | 80,567 | 9,514 | 8,324 |
| 985,723 | 8,213 | 58,769 |  | 8,915 | 59,076 | 5,803 |
| 419 | 47,952 | 9,547 |  | 42,980 | 4,675 | 7,064 |
| 92,035 | 847 | 32,708 |  | 87,209 | 48,063 | 90,568 |
| 87,693 | 36,079 | 4,206 |  | 254 | 76,234 | 7,260 |
| 609 | 6,318 | 58,217 |  | 74,036 | 6,507 | 20,759 |
| 5,812 | 87,024 | 50,679 |  | 492 | 327,684 | 64,028 |
| 23,064 | 3,509 | 6,580 |  | 32,615 | 936 | 2,936 |
| 425 | 24,570 | 42,895 |  | 8,906 | 89,430 | 274,856 |
| 89,712 | 3,615 | 246 |  | 20,853 | 6,079 | 3,072 |
| 3,825 | 67,082 | 75,902 |  | 94,307 | 28,053 | 39,548 |
| 962,578 | 8,234 | 489,362 |  | 798,253 | 4,950 | 76,503 |
| 74,316 | 729,365 | 7,095 |  | 68,905 | 5,713 | 832,946 |
| 8,057 | 5,086 | 653,428 |  | 3,468 | 768 | 794 |
| 59,304 | 80,723 | 2,983 |  | 76,519 | 53,240 | 546,732 |

# Number reading in a student sample of typical readers

We ran the number reading task also for a group of 66 university students, all of whom were native Hebrew speakers, with normal or corrected-to-normal vision, and no reported learning disorders. They were recruited via university social networks. The stimuli, procedure, and error coding were the same as for the two other samples.

The average error rate was 4.2% (SD = 3.04%) – lower than that of the typical readers reported in the main text, who were not exclusively students (unpaired t(236) = 4.404, one-tailed *p* < 0.001).


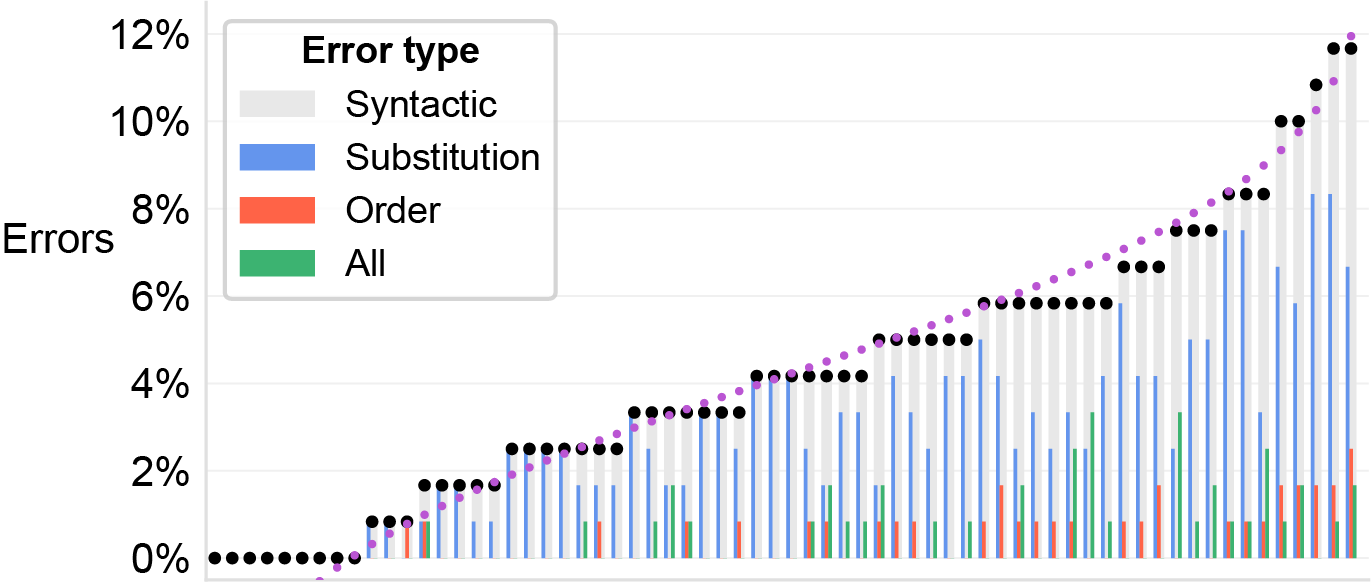


**Fig. S1.** Error rates in the number-reading task. Each participant’s errors are marked by a grey bar with a dot at its top. The internal bars indicate the rate of specific error types. The dotted purple line is the normal distribution for the error rate.


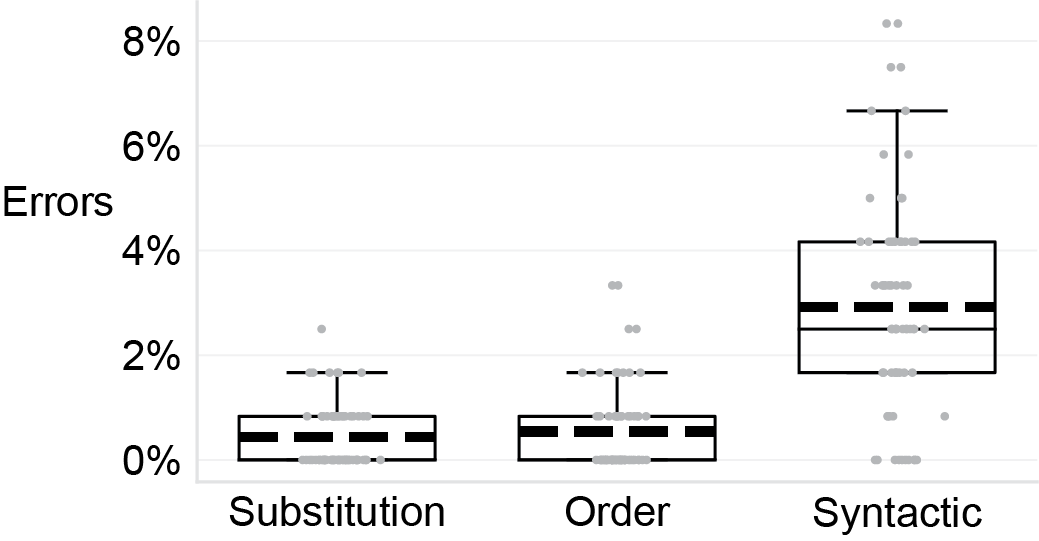


**Fig. S2.** Distribution of error types shows that here too, the syntactic errors were the most prevalent. The boxes show the first quartile, the median, and the third quartile. The whiskers show the 95th percentile. The thick dashed line is the mean. Dots show single participants.

# Distribution of typical readers’ specific error rates

for all error subtypes, the data were well modeled by a normal distribution cropped at 0%, and the fitted distribution captured the data better than the normal distribution based on the observed mean and standard deviation.

**Fig. S3.** Distribution of specific error rates (substitution, order and syntactic errors) in typical readers. Thin grey line shows the histogram of the raw data; black line shows the same data with gaussian smoothing (σ = 2%); dashed purple line shows the normal distribution based on observed mean and standard deviation; and dashed green line shows normal distribution based on mean and standard deviation fitted to data (values provided in figure).
